# The Hospital. Nursing Section

**Published:** 1905-03-04

**Authors:** 


### The Hospital.

### Nursing Section.

Contributions for this Section of "THE HOSPITAL" should be addressed to the EDITOR, "THE HOSPITAL," NURSING SECTION, 28 & 29 Southampton Street, Strand, London, W.C.

No. 962.—VOL. XXXVII. *SATURDAY, MARCH 4, 1905.*

### Notes on News from the Nursing World.

### THE NURSING ARRANGEMENTS AT LINCOLN.

THE account of the arrangements for the nursing of the sufferers from typhoid fever at Lincoln, which is supplied to us to-day by a correspondent on the spot, affords the most convincing proof of the serious character of the epidemic. It will be seen that more than a hundred nurses are now engaged in the city in typhoid cases. Thanks largely to the organising skill, the knowledge, and the energy of Miss Bromhead, the Lady Superintendent of the Institution for Nurses at Lincoln, everything is being done that can be done, so far as nursing the patients is concerned. There has been no lack of nurses from the outset, and, while Miss Bromhead has evidently many willing and useful coadjutors, it is clear that she, as the woman at the helm, has not only a full grasp of the needs of the situation, but acts accordingly.

### A COMMON HOSPITAL UNIFORM.

IN the interview of our Commissioner with the matron of Charing Cross Hospital, whose nursing staff is about to be augmented in consequence of the opening of the new wards, Miss Heather-Bigg states that under existing circumstances she prefers that outdoor uniform should not be worn. But she would be glad if all hospital nurses could wear the same uniform with a distinctive badge, such as the bronze or silver letters worn respectively by the staff nurses and sisters at Charing Cross, and she sees no reason why hospital matrons should not combine to secure such a result. The suggestion is so simple and so sensible that we hope it will be taken up in the spirit in which it is made. There is all the difference between wearing a uniform which is the common property of half-trained persons or nursery-maids, and wearing the recognised uniform of the fully-trained hospital nurse. The addition of the distinctive badge, mentioned by the matron of Charing Cross Hospital, would sufficiently indicate the particular hospital to which the wearer belonged, and outdoor uniform, at present only used for the sake of convenience, would then be worn with satisfaction and pleasure.

### IRISH NURSES AND THE HIGHER EDUCATION MOVEMENT.

A MEETING of the Irish Nurses' Association was held on Friday last to protest against the granting by the Board of Trade of articles of association to the proposed Incorporated Society for Promoting the Higher Education and Training of Nurses. There was a large attendance, and it was resolved that a petition should be sent to the Board of Trade objecting to the incorporation of the society; that the Secretaries of the Royal College of Physicians and Surgeons should be asked to bring the matter under the consideration of the members with a view to lodging objections; that a letter should be sent to each of the seven signatories to the application to the Board of Trade, informing them of the nurses' view of the question; and that full information should be furnished to the Irish members of Parliament.

### MISS PETER.

MISS PAULINE W. PETER, general superintendent of Queen Victoria's Jubilee Institute for Nurses, writes:—"Kindly allow me to correct statements made in your issue of THE HOSPITAL, February 25th. In my letter of regret at not being able to attend the meeting at 20 Hanover Square on 22nd inst. no mention was made either of registration or deputation to Board of Trade. Also your statement as to my being about to proceed to Waltham, Massachusetts, is not correct. I have had no communication with anyone on the subject or as to the scheme now being planned. I am glad to have this opportunity of stating that I am in favour of State registration. Kindly insert this letter in your next issue of THE HOSPITAL." The statement as to Miss Peter and the Board of Trade was made at the meeting referred to. We regret having given currency to the American error, which may be traceable to the circumstance that another English nurse of position with a somewhat similar name is going out to Massachusetts with the object stated.

### POOR-LAW INFIRMARY NURSES AND THE MIDWIVES ACT.

THE Local Government Board have issued a circular to their guardians concerning the case of those persons in the Poor-law service who may be in a position, and who may wish, to prefer a claim to be certified under Section 2 of the Midwives Board. They request the guardians to acquaint any officers in their service whom it may concern that after March 31st no person will be admitted to the Roll of Midwives unless she possesses the qualifications indicated in Section B and C of the rules which include a course of training and the passing of an examination. But those who wish to make application before this rule comes into force should at once write to the secretary of the Central Midwives Board, 6 Suffolk Street, Pall Mall, S.W., who will forward special forms. This notice concerns women claiming admission because they have obtained a certificate of midwifery from the Royal College of Physicians of Ireland, the Obstetrical Society of London, the Coombe Lying-in Hospital, and Gunner's Dispensary, or the Rotunda Hospital for the Relief of the Poor Lying-in Women of Dublin. They must enclose their certificate, or if that be lost a certificate signed by a justice of the peace, minister of religion, a registered medical man, or secretary of an institution approved by the board, that the applicant is the person to whom the certificate was granted. The application must be accompanied by a fee of 10s. It also concerns women claiming admission because they have obtained a certificate from any other institution or examining body other than those specified. They must forward, together with the same fee, the certificate upon which the application is based, together with satisfactory evidence, in the form prescribed by the board (Schedule Form VII.) that before the certificate was granted applicant had received a proper course of instruction and training, (including personal attendance, under competent supervision, upon at least 20 cases during and after labour) had passed an examination in midwifery and the duties of a midwife, and that the examining body who granted the certificate considers the applicant at the present time a proper person to be admitted to the Midwives Roll. Finally, the notice affects women claiming admission because they have been in *bonâ fide* practice for 12 months previous to July 31st, 1902. They must forward a certificate to the effect that the applicant has, to the personal knowledge of the person signing been in *bonâ fide* practice as a midwife for at least 12 months prior to July 31, 1902, and that she is trustworthy, sober, and of good moral character. This certificate must be in the form given in the Schedule (Form IX.), be signed by a justice of the peace, minister of religion, registered medical practitioner, or other person acceptable to the board, and be accompanied by a fee of 10s.

### SCANDAL AT BAKEWELL.

The Bakewell Guardians have a strange conception of the duty which they owe to the public. It appears that a girl of 17 recently left Bakewell Workhouse Infirmary in her nightdress and a blanket at two o'clock in the morning and walked to her home 10 miles across the moors. She had been admitted for medical treatment, and had been locked in a ward with a woman who gave birth to a child during the night. The girl, who is said to have been frightened by the woman's screams, got out of the building through the window. Naturally, the question arises, "Where was the nurse?" and the official story is that the nurse, whose instructions were to make visits every three hours, had not gone into the ward on the occasion of the midnight call, but merely looked through the window. The nurse ultimately arrived on the scene in consequence of the shouts of the male inmates in another ward. In these circumstances it will be learnt without surprise that Mr. Herbert, Local Government Board Inspector, suggested a thorough investigation by a committee. The Bakewell Guardians have, however, decided to disregard his advice, and have calmly declared the incident closed. Is the supreme authority powerless?

### STROUD NURSING ASSOCIATION AND THE COUNTY ORGANISATION.

The proceedings at the tenth annual meeting of the Stroud District Nursing Association were notable for the disinclination shown by the members to affiliate with the new county organisation. It was announced by the chairman that the medical advisers and committee of the Association were in favour of an effort to extend the benefits of trained nursing for the poor throughout the county. But they disapprove of the proposal to recognise and employ, in single-handed charge of districts, nurses who have had but 12, or even but six months' training; the wearing of a new county badge by the nurses of the affiliated Associations instead of, or in addition to, the Association's own badge; and the inspection of the Association nurses and work by the county organisation's superintendent. Several of the speakers who followed expressed themselves opposed to affiliation, the general feeling being that the Association with its excellent nursing home, its capable staff of nurses, and its satisfactory financial position, would only lose by a change which would certainly cause disunion in the ranks of their supporters. In these circumstances the decision to leave things as they are is not surprising.

### GLASGOW NURSES' DANCE.

The Glasgow Nurses' Annual Dance was held in the City Hall last month. There were about 450 persons present, 200 of whom were nurses. Among the matrons were Mrs. Strong, Royal Infirmary; Miss Adams, Ruchill Hospital; Miss Shannon, Western Infirmary; Miss Merchant, Eastern District Hospital; Miss Mosely, Western District Hospital; Miss Simpson, Sick Children's Hospital; Miss McKay, Ear Hospital; and Miss McNicoll, Barnhill Hospital. A great many nurses were in uniform, and the Co-operative nurses looked well in their linen frocks and little scarlet tippets. A number of medical men were among the company.

### ALLEGED ATTEMPT TO POISON A MATRON.

A very serious charge has been made against a nurse at Bath Eye Infirmary. She is accused of having on February 7th attempted to murder Miss Annie Boyd, matron of the infirmary, by administering poison in the shape of a quantity of morphia. The prosecution was not initiated at the instigation of the matron, but the director of Public Prosecution, at the instance of the committee of the infirmary, has taken up the case. So far no direct evidence has been given, and the solicitor to the nurse, a girl of 22, affirms that she denies all the allegations.

### DISTRESSING OCCURRENCE AT A LONDON HOSPITAL.

It of course must often fall to the lot of a nurse, after careful nursing, to lose her patient, but fortunately it is very seldom that death takes place under such distressing circumstances as at St. Mary's Hospital, Paddington, on Thursday night last week. A patient of 29 was admitted for an operation on the right kidney. The operation was quite successful, and the patient would have been discharged in a few days, when unhappily just after the lights had been turned down in the ward, the young woman threw herself out of the window a distance of 60 feet, and was picked up dead. No blame is attached to anyone. The window was protected with five bars outside, but the patient favoured by the dim light had succeeded in reaching it—a distance of 12 feet—opening it by standing on a chair, and, by climbing over the bars, she accompanied her design. The nurse in attendance at the ward heard an exclamation that some one was getting out of the window and rushed to it, but only in time to see the patient disappear. Owing to the pain she had suffered the sufferer had become imbued with the idea that she would never be well again, but there was no reason to suspect suicidal tendency. The matron and the nurse in charge gave evidence at the inquest.

### DISTRICT NURSES IN EAST LONDON.

For the first time the Shoreditch and Bethnal Green Nursing Association held its annual meeting this year in East London instead of West London, and in the evening instead of the afternoon. The attendance on Thursday last week was, however, so poor that the experiment is hardly likely to be repeated. The Mayor of Shoreditch presided, and the report was read by the Rev. H. V. S. Eck, rector of Bethnal Green. He drew attention to the fact that another nurse had been added to the resident staff, making now five staff nurses and seven probationers. This addition had enabled the superintendent to undertake some monthly nursing, which had hitherto been only undertaken under exceptional circumstances. During the past year 1,482 persons were nursed, and 34,205 visits paid. The nurses have all enjoyed long and thorough hospital training, and it was pointed out that when probationers were spoken of, it was not meant that they were learning nursing but that they were learning to apply their knowledge to the altered circumstances of district work. The work of a district nurse, it was affirmed, is far more difficult and requires far more skill than that of a nurse in a hospital or an infirmary. Dr. Knocker spoke of the great poverty among those whom the nurses attended, and said that no one but a doctor could realise what good they did. As a medical man, he could testify to the difference between a doctor acting alone in the homes of the poor, and acting with the co-operation of a district nurse. The improvement was extraordinary, especially in cleanliness and cheerfulness in the home.

### HOW A PARISH PROVIDED A NURSE.

The parish of Emmanuel, Harrow Road, Paddington, has been provided with a nurse in an unusual manner. The vicar, the Rev. Henry Pitt, has introduced a system of free-will offerings. Each person who undertakes to help promises to set aside any sum from a penny upwards and is allotted a number, receiving 52 small envelopes with such number thereon, for the amount to be placed in week by week. These free-will offerings, which are deposited in boxes at the church doors, are supplemental to the church offertories. The scheme has now been in operation for 36 weeks, and the total sum contributed in that period is over £113, the offertories during the time having increased by nearly £30. As one of the direct results of the scheme, a fully qualified nurse to attend on the sick poor of the parish has been added to the staff of church workers. Mr. Pitt has also been able to dispense with the annual bazaar.

### GRAVE CHARGE AGAINST A POOR-LAW NURSE.

At an inquest at Chester Workhouse on Saturday on a woman aged 69 evidence was given by the master of the workhouse, who said that the inmate had her shoulder fractured whilst she was confined to her bed, and that when questioned she stated that a nurse whom she asked to turn her over at first refused, but afterwards got hold of her by the shoulders with both hands, shook her, and struck her on the top of the right shoulder. The medical officer affirmed that death was caused by liver affection accelerated by fracture, but the coroner held that there was not sufficient evidence to send the nurse for trial on a charge of manslaughter, and the jury returned an open verdict. They, however, added that the nursing staff ought to be increased, and that a qualified nurse should be on duty at night. It is, of course, impossible to judge how far the deceased inmate accurately represented the facts of the case, but it is discreditable that in such an institution there should not be a qualified nurse on duty during the night.

### RESIGNATION OF A MATRON.

Nurses who have spent a holiday at Sir Julian Goldschmid's Home of Rest for Nurses, at Brighton, will regret to hear of the resignation of the matron. Mrs. Mackintyre has filled that post for the past 14 years—in fact, ever since the home was opened. Her zeal and endeavours to add to the comfort and enjoyment of the nurses are well known to all who have had the pleasure of staying at the home. She will be greatly missed, and whatever new work she undertakes, her numerous friends will wish her every success and prosperity.

### THE VALUE OF BICYCLES.

In district work the bicycle has become such a valuable ally that we learn without surprise that at the annual meeting of the Leeds and District Nursing Association it was stated that but for the use of bicycles the score of nurses would not have been able to pay nearly so many visits. The number of visits paid last year was 76,589, and the number of cases attended 3,362. The cost per visit was under 7½d. each. We regret that, in spite of this record, the income fell short of the expenditure by the considerable sum of £227. In a city of such great wealth as Leeds this is extremely discreditable, and ought to be speedily rectified.

### SHORT ITEMS.

The name of the secretary of the Hospital for Mothers and Babies and Training School for District Midwives at Woolwich is Miss Alice Gregory. We mention this because an unfortunate confusion has arisen owing to a similarity of surname between Miss Alice Gregory and a Miss Edith Gregory. The hospital at Woolwich, we understand, has not as yet presented any pupils for examination.—The post of matron of Paddington Green Children's Hospital is vacant. The salary is £100 a year. Candidates must be between 28 and 38 years of age, and March 18th is the latest day for sending in applications.—Nurses who wish to go to the Albert Hall during the Torrey-Alexander Mission will be glad to hear that, at the instance of the Ladies' Committee, two boxes are reserved at every meeting for their use, in order that they may be spared the necessity of standing in a crowd outside the building.

### The Nursing Outlook.

" From magnanimity, all fear above;
From nobler recompense, above applause,
Which owes to man's short outlook all its charm."

### THE WORK OF THE INSPECTORS OF MIDWIVES.

The appointment of inspectors of midwives rests with the supervising authorities, whose duty it is to exercise general supervision over certified midwives. In her official capacity the inspector is a public servant under the county council that appointed her, and as such she will have to carry out any orders that may be given to her and obey regulations that may be made for her.

The arrangements and the details of work will necessarily vary somewhat in different counties; as matters are at present she will generally find that she is expected to look to the Health Committee of the council for her orders or else to the medical officer to the council, if there is one. Personally, therefore, the inspector is a link between the Health Committee or medical officer and the midwives. With both of these she will be brought into personal contact and with both of them she must endeavour to be in sympathetic touch so far as is possible. Her success as inspector will largely depend upon the way in which she fulfils this twofold relation; it demands tact and discretion in dealing with human beings, experience or knowledge of varied midwifery practice, and skill in the art of inspecting. A brief sketch of the general outline of the work may serve as an ideal of what these inspections could and ought to be. Having received her orders from the council, or the medical officer, she begins her work of inspection. In doing this, she must be careful not to exceed the powers entrusted to her, or to exercise authority which has not been conferred upon her. With regard to midwifery institutions, she has to remember that inspectors are not permitted to inspect the work of midwives in any hospital, workhouse, or Poor-law infirmary which is under the supervision of a duly appointed medical officer. With this exception, the inspector is entitled to and ought to inspect every midwife's case-book and bag of appliances. She should bear in mind that the ultimate object at which inspection aims is a national one—an attempt to gradually improve the conditions under which childbirth attended by midwives takes place, in order that the health and lives of mother and child may be safeguarded so far as is physically and humanly possible.

The work of the inspector consists in learning whatever (without exceeding her legal powers) she can about the results of the work of the midwife, be they good, bad, or indifferent, and about the conditions under which the midwife's work is carried on. These results the inspector reports to her committee or medical officer, together with any remarks she may deem it right to make, and any information as to causes that seem to explain the results obtained. In her work she will meet with every type and grade of midwife; at the one end of the scale the illiterate, ignorant midwife who scarcely knows how to read or write, much less how to use a clinical thermometer or a catheter; at the other end of the scale, the trained experienced midwife possessed of knowledge and skill in all the latest refinements of midwifery practice. Tact and discretion must therefore be exercised in her interviews with midwives and inspection of their case-books and bags. She should not expect too much from the *bonâ fide* midwife; provided the midwife is "trustworthy, sober, and of good moral character," and the results of her work fairly good, many allowances have to be made for ill-kept books and un-used appliances. In spite of the difficulty of eliciting information from illiterate women who are unfamiliar even with the meaning of terms used in midwifery, the inspector who shows a friendly disposition towards them will, sooner or later, obtain all the information she desires. But she must beware of harshly finding fault, or of getting irritated by these ignorant women; she should seek rather to lead them gently on, encouraging them to habits of greater cleanliness and better keeping of books, by helpful suggestions kindly given. The conditions under which the midwife's work is carried on will be found to vary greatly. In some places the conditions are bad, the mothers she attends poorly clad, poorly fed, and badly housed. In other cases the conditions are far better, and the inspector's skill in inspection will be according to her powers of accurate observation and of testing the accuracy of statements made to her. It is well to be on the alert, and also to note insanitary conditions which could be dealt with by the sanitary authorities. Reforms can only be gradual, but the inspector should always be on the look-out for possible reforms, and should report these to the committee or medical officer.

Personal interviews with midwives and inspection of their case-books and bags may be regarded as the routine work of the inspector. When, however, the work of any midwife is regarded as unsatisfactory, it may be thought necessary for the inspector to inspect the midwife's place of residence and to investigate her mode of practice by attending cases. Before doing so, it is advisable that the inspector who is new to the work should ask the committee or the medical officer under whom she serves whether it is their wish that she should use her own judgment in this matter, or whether they wish her to report to them the unsatisfactory nature of the midwife's work and await further instructions.

In inspecting bags, it is the inspector's duty to note whether proper cleanliness is being observed and whether the appliances are in good condition and fit for use. It is not, however, her duty to insist that the midwife shall use a glass catheter rather than a soft catheter or a soft one rather than a glass one, or even that she shall use a catheter at all. With regard to appliances, practice varies somewhat in different places; some nurses and midwives have been taught to use soft catheters, others have been taught to use glass catheters. The essential thing is that the catheter be clean and, if a glass one, unbroken. The same remarks apply to other usages. Moreover, in the clumsy hands of an untaught *bonâ fide* midwife the use of any appliances for the mother and of antiseptic lotions for cleansing the eyelids of the newborn child may be sources of danger, and it is better that such a midwife should not use these at all than that she should use them and do harm thereby.

### Lectures on the Nursing of Sick Children.

By James Burnet, M.A., M.B , M.R.C.P.Edin., Registrar, Royal Hospital for Sick Children ; Clinical Tutor, Extramural Wards, Royal Infirmary ; and Physician to the Marshall Street Dispensary, Edinburgh.

### I.—SOME GENERAL POINTS.

The management of sick children differs in many essentials from that of adults who are ill. Children do not give us much help with regard to their ailments. Younger children are constantly fretful when sick, and it is not always an easy matter to make out why they are peevish and cross. The nurse who makes sick children her life's work must be gifted with more than ordinary patience. Her temper must not be easily ruffled, and under all circumstances she must be kind and gentle towards those little ones who are, for the time being, under her immediate charge.

In these lectures I shall try to be brief and to give those essential facts which it is so necessary to bear in mind during everyday work in the wards, or, it may be, in the private house by and by. I would advise my readers that a summary of each lecture be made in a small note-book, which can be carried in the apron pocket and referred to at odd moments. In this way a number of useful facts will be ready at hand to be made use of when occasion arises.

### How to Take a Sick Child's Temperature.

I always judge of a nurse's skill from the accuracy with which she uses the clinical thermometer. It is often the case, is it not, that the nurse looks upon temperature-taking as a bore, and as a piece of routine which is not worth paying much heed to? This is a great mistake. The temperature chart is a very important matter to the medical attendant, and it is a comfort to him to know that the nurse can be absolutely relied on to take the temperature accurately.

Always see that the thermometer is shaken before being used. The index should always point to the figure 95 on the scale before the thermometer is inserted. Five minutes is usually required for the accurate taking of temperature. The instrument should be placed in the groin, and not in the mouth or axilla. There is great danger lest it be broken if placed in the mouth, and children usually resent the arm being held to the side, which is necessary when the thermometer is placed in the axilla. Some physicians require that the temperature be taken by placing the thermometer in the rectum. When this is so the bulb of the instrument must previously be oiled or smeared with vaseline. The child is then turned on to its side and the thermometer passed up into the bowel a distance of three inches, and the buttocks pressed together over it.

After use the mercury is to be shaken down again, and the thermometer cleansed, dried, and returned to its case. Remember that very little will cause the temperature of a young child to rise. Often slight derangement of stomach or bowels will send the index up to 104°. So, too, with trivial throat conditions. At the same time, whenever you find the temperature above normal, the medical attendant should be at once informed and the cause sought.

### How to Feel a Child's Pulse.

It is of no use to take a child's pulse while he is crying or after a fit of coughing or vomiting ; take it during an interval of rest. The pulse is best taken while the child is asleep. This can often be done by placing the finger on the temporal artery just at the side of the head. If it is taken at the wrist two fingers should be placed—the first and second of the right hand—over the artery. Count the beats for four successive quarter minutes by the watch, and note if the number of beats is the same in each quarter-of-a-minute interval. If not, then it is certain that the pulse is irregular, and the fact should be noted and reported. In infants the normal pulse-rate varies from 100 to 120 per minute, and anything over this would be termed a rapid pulse. At the sixth year anything over 90 is abnormal, and at the tenth year the pulse should not be over 85.

Remember that an irregular pulse is often quite normally met with during infancy while the patient is asleep; but if it falls below 100, and is at the same time irregular, some serious mischief should be suspected. A slow pulse in older children who are obviously ill may mean inflammation of the brain.

### How to Take the Respirations.

Never on any account place a hand on the patient's chest. It is sure to disturb the child, and he will either cry or become restless, thereby causing him to breathe more rapidly. It is best to watch the movements of the chest while the patient is asleep. They can easily be counted then by his cot-side. Do not be satisfied with a one-minute's count, but go on taking the respirations for three or four successive minutes and then take the average. This method will give more accurate results than mere reliance on a one-minute count. Never attempt to take the respirations after the child has been crying, vomiting, or tossing about. Remember that during the period of infancy—that is up to the second year of life—the respiration rate varies between 25 and 35, and from the second to the tenth years the rate is usually between 25 and 20. If the nostrils dilate there is some disturbance of the breathing ; and, indeed, a restless baby's respirations may often be counted by watching the movements of the nostrils when it is impossible to count those of the chest.

### How to Give Medicines.

Children, like adults, have their likes and their dislikes; but as a general rule they hate medicine, even when its taste is not unpleasant. Some children will take castor oil readily, though they will refuse syrups ; while others again will take cod-liver-oil emulsion, though they absolutely refuse to take peppermint water. It is important in giving medicines that they should be made as pleasing to the palate as possible. A little glycerine added to a nauseous draught will often help the patient to get it over. It is not, as a rule, advisable to put medicines in the food, as the child may eventually find this out and refuse even his milk and soups. Powders of small size may be smeared on the tongue and some milk or water given to the child to drink; or the powder can be mixed up with a little syrup or jelly in a teaspoon and administered in this way. When a child refuses to take his medicine, get someone to hold his head while the spoon is pushed well to the back of the patient's tongue. If the mouth remains closed it can usually be opened by holding the child's nostrils or by insinuating the handle of a teaspoon at the side of the mouth. See that the child really swallows the medicine. Very often even quite young patients will hold it in the mouth for some minutes and then spit it out again. It is never a good thing to use too much force with a child who is dangerously ill, but as a general rule the patient should be coaxed or compelled to take his medicine by one or other of the plans I have mentioned. Preparations containing opium must be given with great care to young children, as they are very easily brought under its influence. When such preparations are ordered never wake up the patient to give him the medicine; nor should the dose be repeated, if the child seem at all drowsy, without first obtaining the sanction of the physician in charge of the case.

I have tried in this short lecture to give some useful facts. In my next lecture we will consider the feeding of sick children.

### The Nurses of Charing Cross Hospital.

### INTERVIEW WITH THE MATRON. BY OUR COMMISSIONER.

A LITTLE more than two years ago Miss Mildred Heather-Bigg succeeded Miss H. A. C. Gordon as matron of Charing Cross Hospital, in which institution she herself served as sister of the Golding Ward after her training at University College Hospital. In the interval, however, she was for seven years matron of Chelsea Hospital for Women, and, accordingly, entered upon the duties of her new appointment equipped by ripe experience as well as by training. Short as her period of office at Charing Cross Hospital has been it has been full of incident, and when I called upon her the other day to ask in detail how the nursing arrangements have been affected by recent changes, she referred to one of far-reaching importance.

### FOUR YEARS' TRAINING.

"The most noteworthy change since I have been matron here," said Miss Heather-Bigg, "is the extension of the time of training to four years."

"Of course, you consider that the extension is an advantage to the hospital?"

"I consider it good for the nurse and good for the hospital. The nurse gets more experience. It is true that here, even in the first year, there are no brasses to polish, no floors to scrub, no window-sills to clean. The first year probationer has only to dust in the wards. But everything is new to her. She does not, at the outset, possess the capacity for picking up what she has to learn. In the second year she can act more on her own responsibility. She is put on night duty, or on an operation case, and has to take notes. I should like to emphasise the value of note-taking. In private nursing it is a great thing to be able to write clearly. Our second-year nurses summarise at the end of the day the amount of sleep a patient has taken, the amount of food received, and any great change of temperature. In fact, the salient points of the case are all put down. In the third year the probationer works round the wards. If the senior nurse happens to be ill, she takes the duty, and she wears the silver badge 'S.N.' to show that she is temporarily as staff nurse in charge of the ward."

"And the fourth year?"

"During the fourth year she has holiday duty given to her, and is invested with responsibility for the ward at night. I may say that, as far as possible, half the time of the probationer in her third year is spent in the medical and half in the surgical wards, so that when there is a vacancy for a sister, the staff nurse may be eligible for it at the end of the fourth year."

### PROMOTION FROM WITHIN.

"The principle in force being promotion from within?"

"Yes. By the close of this year we shall not have to take in any sisters from outside. When I came I had necessarily to take some in, but our system now is to train up the nurses by degrees in order to fit them to eventually become ward sisters. We consider it is desirable from every point of view, that our sisters should all be Charing Cross nurses."

"In what respect do you think the hospital benefits by the addition of the fourth year to the period of training?"

"Instead of the nurse leaving at the end of the time when she is really qualified, she stays on and gives the hospital the benefit of her training and experience. She then understands ward management, is able to be of general use, and is ready to take a sister's post."

### THE EXAMINATIONS.

"It would be interesting if at this point you would tell me something about the examinations?"

"During the first year the probationers attend a course of 10 lectures on anatomy, and 10 on elementary nursing, delivered either by the matron or the home sister, and there are 20 practice classes in bandaging. At the end of the first year an examination is held, and those who fail are allowed to try a second time six months later. The second year nurses attend a course of 10 lectures on anatomy given by a surgeon of the staff, and a course of 10 lectures on physiology given by a physician. Ten lectures are given to the third year nurses on the dangers and treatment of medical diseases, and a course of 10 lectures on surgical diseases and their treatment. As to examinations, the second year nurses have a class examination only, but the third year nurses are examined in all that they have learnt during the three years. If they fail, they, too, are afforded another chance of passing at the end of six months."

### COOKERY LECTURES AND PRIVATE NURSING.

"I believe that under your auspices cookery lectures have been introduced?"

"Yes; but we are indebted to the National School of Cookery, by whose representatives the lectures and lessons in cooking are given gratuitously. The course is attended by first and second year nurses. At the end of the course they have to pass two examinations. The examiners set questions, and the nurses have to cook before them. First and second class certificates are given them according as they succeed in what they have been taught. In my opinion the practical knowledge of cookery is extremely useful to nurses, particularly to those who go in for private work. For instance, our probationers are taught to prepare just the dainty dishes which are needed for an invalid, including an omelette, or a soufflé; how to grill a sole; how to cook tripe, which is now so much recommended by medical men; and how to make a wine jelly. The nurses receive three months' training in housekeeping.

### THE NURSES OF CHARING CROSS HOSPITAL—*Continued.*

### An Increase in the Staff.

"When does your next examination of the third-year nurses take place?"

"The second week in March. It is, of course, the first under the new system. There are 12 candidates, and I hope that the majority, if not all, will pass. I shall select the best for the new wards, which will be opened in March."

"How many more nurses shall you need?"

"Eight, six of whom must be staff nurses. There are six wards, with 90 beds in the new surgical block for men and women; but three of the old wards will be closed for a time. When both the hospital and the nurses' home are entirely completed, the nursing staff will be augmented to 95, and this will involve the appointment of three more sisters, as well as several more probationers. The existing staff consists of 50 probationers and staff nurses and 12 sisters, and in March we shall increase the 62 to 70."

### The Value of Fire-Places.

"We gave an account of the Home at the time it was opened, and of course I knew that each nurse had a separate room. I also notice that in each room there is a fire-place. Do you prefer it to a radiator?"

"I think that it is very much better. A fire-place is a source of ventilation, and it is a great comfort and convenience. When a nurse is unwell but not ill enough to be sent to the sick-room, she has a fire in her own room, which would be very cheerless without it. Moreover, I think that, while radiators are very useful for warming the passages, heating by hot air in the bedrooms is enervating."

"In going over the Home I observed on one of the doors of the bath-rooms at the end of the corridor the word 'Engaged.' Does that mean that the doors are not locked or bolted?"

"Yes. And by this arrangement if a nurse feels unwell in the bath-room and rings the emergency bell, some one is able to go at once to her assistance. Speaking of the emergency-bell, the fire-bell is rung every night at 10.15, so that we can tell whether it is in working order. Hydrants, which the nurses have been taught to use, are attached to the main. As you see, at the end of every corridor there is a bridge which communicates with the ward opposite. In the event of fire a bell in the matron's room rings violently, so that I should get the alarm at once. In fact we have taken every precaution against fire."

### The Work in the Wards.

"How are the nurses allocated in the wards?"

"There are 22 beds and two cots in a ward, with a sister in charge, a staff nurse, and two to three probationers. In the theatre there are always two sisters, and a senior and junior probationer on duty. As far as we can, we arrange for each probationer to have four months in theatre during her training. The ward sister looks after the sponges, and prepares the dressings for the surgeon; the theatre sister prepares the instruments and ligatures, while the two nurses and probationer attend to all the minor details. The object is to make everything as aseptic as possible. Then, in the out-patient department, there are a sister, a staff nurse, and two probationers; and there is a staff nurse in charge of the electrical department."

"Are there any special features in the hours of duty?"

"The probationers and staff nurses rise at 6, attend prayers at 6.30, breakfast at 6.35, are in the wards at 7, dine at 12.45, have tea at 4.30, supper at 8, attend prayers at 8.30. Half-an-hour is allowed for each meal (dinner three-quarters of an hour), and bed-time is at 10. Off-duty time is from 10 to 12 or 2 to 5, one day a month, and the yearly holiday is three weeks. The night nurses rise at 7.45 P.M., attend prayers at 8.30 P.M., breakfast at 8.35, are in the wards at 9 P.M. and off at 9 A.M. Dinner is at 9.30 A.M., bed at 1 P.M., and time off 10 A.M. to 12.30 P.M. Each day nurse is allowed to be off duty for a quarter of an hour before 10 A.M., and each nurse on night duty is given 20 minutes for a midnight meal."

### Night Duty and Diet.

"How many are on night duty?"

"Eight in addition to the night sister, andthere are also two extra nurses who go into the wards to relieve the nurses when they take their midnight meal."

"When do the sisters go on duty?"

"At eight and they are off duty at nine. They have a month's holiday, and their salaries begin at £30, and rise, by increments of £5 a year to £45. The probationers, who must be between twenty-three and thirty, are not paid the first year, but the second they receive £15, the third £20, and the fourth, on passing qualifying examination for staff nurses, £26."

"You have a certain number of special probationers?"

"You mean those who pay. Yes, it meets the requirements of some parents to send their daughters for a year. The fee is 52 guineas, and the paying probationers have to provide their own uniform and laundry. If they stay on they are placed for the rest of the four years on the same footing as the other nurses.

### A Common Hospital Uniform.

"Outdoor uniform is not prohibited?"

"No; we provide indoor uniform to all the ordinary probationers, but under existing circumstances I prefer that outdoor uniform should not be worn. At the same time, if it could be agreed that all hospital nurses should wear the same uniform with a distinctive badge, such as the bronze or silver letters worn indoors by our staff nurses and sisters respectively, I should be glad."

"Do you think that the matrons could be got to combine?"

"Why not? When I first went into nursing only hospital nurses wore outdoor uniform. Then private nurses started it, and finally nursery maids and other persons who discredited its use. But if hospital nurses could be distinguished by their particular uniform the position would be altered. The surgical homes have done much to bring uniform into contempt."

"You would have them registered?"

"Certainly, and inspected also. They employ a number of half-trained nurses. I am in favour of the registration of nurses because I believe that it would put an end to the employment of such persons. The public, I think, would then be able to judge whether they have value for the fee they pay. Within my own knowledge there are surgical homes which take nurses with special training only and send them out to nurse typhoid, charging two guineas a week for their services. Scandals of this kind could not continue under a system of State registration.

### Presentations.

West Derby Infirmary, Liverpool.—Miss L. R. Jones, on leaving West Derby Infirmary, has been presented by the staff with a silver mounted brush and comb, hand mirror, and button hook.

### Lincoln Typhoid Epidemic: The Nursing Arrangements.

SPECIAL ARTICLE.

DURING the present outbreak of typhoid in Lincoln, the organisation of the district nursing, and the nursing and most of the arrangements in the temporary typhoid hospitals have been placed entirely in the hands of Miss H. Bromhead, lady superintendent of the Institution for Nurses, Lincoln.

### THE HOSPITALS AND STAFF.

In December 1904 she was requested by the sanitary committee to open the hospital for typhoid erected by herself some time ago for the town, and supplied gratuitously with nurses from her institution. This hospital is an iron building containing 11 beds, nursed by five nurses. The first patient was admitted on December 7th, and in a short time all the beds were filled. On January 29th in the present year the chairman of the Lincoln Sanitary Committee requested Miss Bromhead to provide at the expense of the Corporation extra district nurses for the typhoid patients in their own homes. On the same date sanction was obtained to open the City Scarlet Fever Hospital for typhoid patients only. This holds 25 beds, and nine nurses were provided by Miss Bromhead for it. These arrangements were speedily followed by the decision of the Sanitary Committee to fit up temporary typhoid hospitals throughout the town, in halls, mission chapels, etc., to be rented for that purpose. To the medical officer of health for the City of Lincoln, and to Miss Bromhead and the sanitary inspector, were entrusted the entire arrangements for the fitting up of, and the conversion of these buildings into temporary hospitals and the housing and catering for the nurses. On February 2nd the first temporary hospital was opened in the Blenkin Memorial Hall, with 17 beds and a staff of seven nurses. On February 7th the second hospital at Lemport Hall, with 10 beds and five nurses, also a third hospital, the Vernon Street Mission Chapel with 20 beds and eight nurses. By this time the cases had so rapidly increased, and the demand for more hospital accommodation was so urgent, that the Board of the Lincoln County Hospital decided to at once reserve one ward of 20 beds for typhoid cases for the use of the corporation. Leave was also obtained from the authorities to hire the large Volunteer Drill Hall. On February 9th this was opened, with 100 beds, and a sister, Miss Sheppard, late staff nurse at King's College Hospital in charge, a staff of 34 nurses, several wardmaids, and a cook. It is divided into two wards, kitchen, etc., nurses' dining-room, and various storerooms. There is sleeping accommodation on the premises for 17 nurses and the sister-in-charge—17 nurses are housed near in the houses of various friends. The nurses' meals are provided by a caterer and brought to the Drill Hall, the kitchen arrangements there being only adequate for the patients. On February 22nd St. Martin's parish-room was opened as a hospital with 22 beds. This when filled will have a staff of 10 nurses. Each hospital has its own doctor—local medical men living within easy reach, and all the hospitals are supervised by Dr. Harrison, Medical Officer of Health for the City of Lincoln. At the smaller hospitals are appointed nurses-in-charge, and at the Drill Hall Miss Bromhead has placed a sister-in-charge. Two horse ambulances are being used for the removal of patients to hospital, one single and one double. An ambulance nurse attends each removal.

### DISTRICT NURSING ARRANGEMENTS.

For the patients unable to be received into hospital Miss Bromhead has arranged the town in districts, the nurses being, as far as possible, in the centre of each. Twenty-one nurses are employed in this way. The districts are divided into two groups, one group reporting to Miss Bromhead and the other to a deputy, one of the clergy of Lincoln, who has done splendid work by helping to organise in one part of the town. All the 70 nurses of Miss Bromhead's own nursing institution being fully engaged with the usual private work and district nursing, she has been obliged to draw largely upon other institutions throughout England. In all she is employing 96 extra nurses—81 paid for by the Lincoln Corporation, 12 supplied gratuitously from her own institution, two given by the Blackheath Nursing Institution, and one given by a friend. In addition to these Messrs. Ruston and Proctor and Messrs. Clayton and Shuttleworth have undertaken to provide district nurses for all their work-people, and several ladies have provided a few night nurses. Each day all the notifications of the cases are sent to Miss Bromhead from the Corporation offices. A district nurse is sent as soon as possible to each if needed. The cases have not been restricted to one or two parts of the town but all over it, this has made the district nursing unusually heavy.

### THE KINDNESS OF HOUSEHOLDERS.

The housing of the nurses, district and hospital, and the catering, has been to a great extent helped by the kindness of many householders who have taken in a large number of nurses to sleep and, in some cases, for meals. For the feeding of the district patients 11 depôts have been established in the town where Benger's food, beef-tea, milk, brandy, etc., are supplied on receipt of an order signed by doctor or nurse. Miss Bromhead has been enabled to supply these depôts not only with the foods, etc., just mentioned, but with bed linen, clothing of every description, and every possible comfort, by the great kindness of very many friends who most generously are subscribing to her Fund for Comforts for Typhoid Patients. Things have literally poured in, so much so that she has been able to help the hospitals with extra linen, much of which was very badly wanted, as well as doing the same for all the district patients. Supplies of every kind as well as money have been received through the goodness of sympathisers. In this account of the hospital and general nursing arrangements it is not, of course, possible to deal with the many other cases of typhoid now in Lincoln being nursed by Miss Bromhead's private nurses and by those of other institutions.

### Training at a Nursing Institute.

AT the Brompton County Court last week, Miss Minnie Quest, a nurse, brought an action against Miss Elizabeth Margaret Simmons, proprietress of the Fulham Nursing Institute and Midwifery Training School, claiming £50 in respect of an alleged breach of contract.

Plaintiff's solicitor stated that his client came in communication with the defendant through an advertisement for midwifery pupils. It appeared that the defendant's fees were £13 13s. for three months' training, with £1 1s. for the L.O.S. examination. Eventually, however, the defendant agreed to take the plaintiff at an inclusive fee of £10 10s. The girl arrived at the home on November 30th last, and in the course of the first conversation she said to the defendant, "Of course you are a trained nurse and midwife yourself?" and the reply was "No, I should hate the work!" The plaintiff thereupon asked who trained the pupils, and defendant said, "I have a proper trained midwife, but she is away at present." Subsequently three different nurses gave plaintiff some instruction. Just before one of the Sisters left, the defendant told plaintiff to copy all the notes of lectures in the Sisters' books, and that she (defendant) would teach her herself. The plaintiff strongly objected, remarking, "Why, you don't know as much as I do, and that is very little." Other differences arose, and at half-past one on a Sunday morning the defendant sent the girl off.

The plaintiff bore out solicitor's statement. In cross-examination she denied having broken any of the rules of the establishment, or being rude to the defendant.

Two nurses who were at the home the same time as the plaintiff said that they always found plaintiff well behaved and obedient to the rules of the establishment.

The defendant stated that she had conducted the establishment since November 1903, and during the first twelve months had twelve pupils. The plaintiff received proper lessons, and never complained about the nature of the instruction. She had occasion almost every day to complain of the plaintiff's conduct. The girl was late at meals, and wore dirty aprons, kept on her outside boots after coming into the house; went out without permission, and was generally disobedient.

Dr. John H. Griffiths, Munster Road, Fulham, stated that he had from time to time given lectures to the students of the home, and that he had always found the plaintiff well-behaved.

The jury returned a verdict in favour of the plaintiff, assessing the damages at £5. The Judge, in giving judgment, said he thought the verdict a very proper one, and allowed costs.

### Central Midwives Board.

A meeting of the Central Midwives Board was held at the Board-room on Thursday last week. All the members were present except Miss Wilson, who was prevented by illness from attending.

A number of letters were read and dealt with. These included one from a midwife, asking "how to act in case of a refusal to attend on the part of a registered medical practitioner, sent for under Rule E 17." The writer said that some doctors expected fees of from three to five guineas "brought to their doors." The board decided that the local supervising authority should settle such a question, and that their attention should be drawn to the circumstances; a copy of the letter to be sent from the board to the Privy Council.

The applications of 2,400 midwives for certificates were approved, bringing the total number now on the roll up to 15,490.

The board then considered the report of the standing committee which dealt with cases of alleged misconduct on the part of various midwives. In several instances no action was taken; two applications for certificates were refused. Two certified midwives against whom *primâ facie* cases of misconduct had been established in the opinion of the Local Supervising Authority were cited to appear before the board on March 16th. It was agreed that a Lancashire midwife, who had been proved to have caused the death of an infant by cutting its tongue, should be severely censured.

A resolution moved by Miss Paget,

"That no institution with less than 75 deliveries annually shall be approved as a training school"

was passed unanimously. Miss Paget, in support of the motion, said that she thought this principle having been adopted by the board in reference to Poor-law institutions should also be extended to others. It should not inflict injury upon smaller institutions. In the case of small maternity charities, other than Poor-law, if considered suitable by the board, the head midwife can be approved for signing Forms 3 and 4, and this midwife, unlike the official under the Poor-law, would be subject to the rules of the Central Midwives Board (Section E).

### Appointments.

[No charge is made for announcements under this head, and we are always glad to receive, and publish, appointments. The information, to insure accuracy, should be sent from the nurses themselves, and we cannot undertake to correct official announcements which may happen to be inaccurate. It is essential that in all cases the school of training should be given.]

Adelaide Hospital, Dublin.—Miss Rachel Burkitt has been appointed night superintendent. She was trained at Sir Patrick Dun's Hospital, Dublin, where she was afterwards sister-in-charge of the gynæcological department and of the surgical wards, operating theatre, and casualty department.

Ancoats Hospital, Manchester.—Miss Mary Hull has been appointed sister of the children's ward, and Miss Annie Roy night sister. They were both trained at the Ancoats Hospital.

Brentford Union Infirmary, Isleworth.—Miss Edith A. Hood has been appointed night superintendent. She was trained at Birkenhead Infirmary, where she has also been sister. She has since been sister at Bethnal Green Infirmary. She holds the L.O.S. certificate.

Bromsgrove, Droitwich, and Redditch Hospital.—Miss Sadie C. G. Barrs has been appointed matron. She was trained at St. George's-in-the-East Infirmary, London, and has since been sister at the Borough Hospital, Plymouth, and charge nurse at Clock House Hospital, East Ham.

Bury Infirmary.—Miss A. West has been appointed charge nurse. She was trained at the City Hospital, Nottingham, and Swansea General Hospital. She has since been sister at the Royal Portsmouth, Hospital.

Cottage Hospital, Ulverston.—Miss Clara Reynolds has been appointed matron. She was trained at Retford Hospital, and has since been staff nurse at Derby Royal Infirmary, and charge nurse at Retford Hospital. She has also done private nursing in connection with the Bradford Institute and St. Mary's Home, Ulverston.

General Hospital, Weston-super-Mare.—Miss Clara S. G. Brice has been appointed staff nurse. She was trained at the Infirmary, Fishponds, Bristol, where she has since been staff nurse. She holds the L.O.S. certificate.

Holborn Union Schools, Mitcham.—Miss Amy Sayer has been appointed assistant nurse. She was trained at the Alexandra Hospital, Bloomsbury.

Jessop Hospital for Women, Sheffield.—Miss Florence Whelpton Johnson has been appointed staff nurse She was trained at the Walsall and District Hospital, where she has since been sister.

Leicester Borough Hospital.—Miss Catherine Marie Duffy has been appointed matron. She was trained at the Royal Albert Edward Infirmary, Wigan. She has since been head nurse at the Park Hill Hospital, Dingle, Liverpool; night superintendent and assistant matron at the New Lodge Moor Hospital, Sheffield.

Richmond, Whitworth, and Hardwicke Government Hospitals, Dublin.—Miss Eileen O'Clery has been appointed sister. She was trained at Sir Patrick Dun's Hospital, Dublin, where she was afterwards sister-in-charge of the children's ward and of the gynæcological department.

### Echoes from the Outside World.

### Movements of Royalty.

The King and Queen held the second Court for the season at Buckingham Palace on Friday night. Among the *débutantes* was Princess Ena of Battenberg, who was presented by her mother, Princess Henry. She wore a dress of white satin and tulle wrought with silver.—On Saturday the King and the Prince of Wales were present at a football match played at the Queen's Club ground at West Kensington. During the day his Majesty and the Queen visited the Royal Academy in order to inspect the Watts collection, and in the evening the King gave a dinner-party at Buckingham Palace.—On Monday afternoon the King left London for Portsmouth in order to pay a visit to Rear-Admiral Prince Louis of Battenberg, commanding the Second Cruiser Squadron. His Majesty occupied the Rear-Admiral's quarters on board, which, though somewhat circumscribed, are very comfortably arranged. In the evening a number of officers were commanded to attend the dinner.—On Tuesday the King inspected the ships, and after the inspection there was a parade. In the afternoon he returned to London.

### Visit of the Prince and Princess of Wales to India.

The King has approved arrangements for the visit of the Prince and Princess of Wales to the Indian Empire. It is expected that their Royal Highnesses will arrive in India during November, and that they will remain there until March 1906. They will be received by the Viceroy as the representative of his Majesty. It is their intention to visit, so far as the time at their disposal will permit, the principal cities of British India and the more important native states. During the tour the Prince of Wales will receive the princes and chiefs who rule under the paramount protection of the King Emperor, and will hold levées at which will be presented to him the principal personages of his Majesty's Indian dominions. The King, on the advice of the Government, after consultation with the Viceroy, has been pleased to direct that the exchange of ceremonial presents on the occasion of the visit of their Royal Highnesses shall be dispensed with. The last Royal visit to India was in 1875 when the present King as Prince of Wales made a tour of some months. On that occasion ceremonial presents were exchanged to the value of many thousands of pounds.

### Betrothal of Princess Margaret of Connaught.

It was officially announced on Saturday that the King and Queen had received the gratifying intelligence of the betrothal of Princess Margaret Victoria Augusta Charlotte Norah of Connaught, to Prince Oscar Frederick William Olaf Gustavus Adolphus, Duke of Schonen. Princess Margaret is the eldest of the three children of the Duke and Duchess of Connaught and was born at Bagshot Park on Jan. 15, 1882. She is a Lady of the Royal Order of Victoria and Albert and of the Imperial Order of the Crown of India. She is especially fond of country life and sport. Prince Oscar is the eldest of the three sons of the Crown Prince and Princess of Sweden and Norway and was born at Stockholm on Nov. 11, 1882. His mother is the daughter of the sister of the late Emperor Frederick of Germany.

### The North Sea Inquiry.

The International Commission held its final sitting in Paris on Saturday afternoon, and Admiral Fournier, the presiding commissioner, read the report. The chief points of the decision, which is divided into 17 heads, are that the Russian Admiral Rozhdestvensky was justified by warnings in taking every precaution; that the delay to the transport *Kamschatka* was, perhaps, the incidental cause of the affair; that firing was opened in consequence of the appearance of a "suspicious vessel"; that the responsibility for this act and its consequences must fall on Admiral Rozhdestvensky; that the Russians fired on their own vessel, the *Aurora*; that the fishing fleet committed no hostile act; that no torpedo boats were present, but that the Commission thinks the Russian sailors were under an "optical illusion"; that the firing was unduly prolonged, and that the matter should have been reported to British or French territories so that aid could have been sent; and that the Admiral and his staff showed no want of courage and humanity. At Hull, and in fact all over England, the result is regarded as a great triumph for the cause of arbitration.

### New Cathedral in Berlin.

On Monday the new Cathedral in Berlin was dedicated with much ceremony in the presence of the German Emperor and Empress, many Royalties and representatives of Sovereigns and Churches of the Protestant States of Europe. Prince Arthur of Connaught represented King Edward, and the Bishop of Ripon was also present. The new edifice, a basilica, is built of Silesian grey sandstone in the style of the Italian Renaissance, has been erected at a cost of £500,000. The central cupola with the cross which surmounts it reaches a height of about 374 feet or over 9 feet more than the height of St. Paul's Cathedral in London. The organ has 114 stops and is the largest in Germany.

### The Whistler Exhibition.

The exhibition of Whistler's works now open at the New Gallery is wonderfully representative. The organisers have been particularly fortunate in having been allowed to borrow about a couple of hundred of his etchings from the King's Library at Windsor, and also one of Whistler's masterpieces, "Portrait of My Mother," from the Luxembourg Gallery. The portrait of "Carlyle," sent by the Corporation of Glasgow, naturally occupies a place of honour. Other celebrated pictures are "The White Girl," which went years ago to America, and has now returned for the first time for exhibition; "Miss Alexander," wherein the white and silver-grey with its touches of black form such a charming setting to the portrait of the fair little girl; and "Sarasate," which, now that it is seen again, seems to reveal fresh beauties we had almost forgotten. "St. Mark's" at Venice will be one of the pictures most discussed because, though the façade has been painted thousands of times by day, few have succeeded, as Whistler did, in painting it by night. Another night effect which should not be overlooked is "Battersea Bridge" by moonlight. On Saturday the Whistler Exhibition was attended by 2,927 persons, a record which exceeded by nearly 1,000 the largest attendance in any single day at any previous exhibition held in the New Gallery.

### New Play at the Avenue Theatre.

Mr. R. C. Carton's latest play, "Mr. Hopkinson," is a great success. The author calls it a farce, but the word 'comedy" would perhaps more aptly describe the doings of the hero, a greasy-haired, oily-mannered, vulgar *parvenu*, who, from being a book-keeper in a suburban shop, comes into a large fortune and goes into "Society." The sketch is in no way over-coloured, and Mr. Carton is probably drawing from life, for such persons as Mr. Hopkinson are known to most of us, though they are not always equally amusing. Mr. James Welch pourtrays the character as if to the manner born, and has never been seen to better advantage. He is admirably supported by Miss Annie Hughes, who makes much of the small part of the milliner with whom as a shopman he had tender passages, and other members of the company.

### Notes and Queries.

**REGULATIONS.**

**The Editor is always willing to answer in this column, without any fee, all reasonable questions, as soon as possible.**

**But the following rules must be carefully observed :—**

**1. Every communication must be accompanied by the name and address of the writer.**

**2. The question must always bear upon nursing, directly or indirectly.**

**If an answer is required by letter a fee of half-a-crown must be enclosed with the note containing the inquiry.**

*Treatment of Hair.*

(163) Can you give me the address of the hospital in London which is for special treatment of the hair alone ?—*A. E. S.*

There is no hospital for treatment of the hair alone. But a hospital for skin diseases would deal with diseases of the scalp and hair. There are in London :—St. John's Hospital for Diseases of the Skin (out-patient department), Leicester Square, W.C.; The Western Skin Hospital, 179 Great Portland Street, W.; The Hospital for Diseases of the Skin, 52 Stamford Street, Blackfriars, S.E.

*Male Nurses.*

(164) Will you kindly let me know if there are any hospitals in London where male nurses are trained ?—*D. R.*

Male nurses are received as probationers for two years' training at the National Hospital for the Paralysed and Epileptic, Queen Square, W.C. Write to the matron.

*Recognised Training School.*

(165) I am under the impression that a hospital of this size (60 beds) is not a recognised training school. Will you kindly tell me whether I am right about this ?—*E. R. D.*

A recognised training school should contain not less than 100 beds ; and, of course, there are other qualifications.

*Army Nurse.*

(166) I am 18 years of age and very much want to become an Army nurse. Can you tell me if I am old enough and where I should apply ?—*M. M.*

Army nurses must not be under 25 years of age, and must have had three years' training in a recognised hospital. You are too young to become a probationer excepting in a children's or some special hospital. "The Nursing Profession : How and Where to Train," would help you a great deal.

*Note-book.*

(167) Will you kindly inform me whether there is a book on "How to Take Note" when attending nursing lectures, and what books would be best to study during a period of waiting ?—*Anxious.*

We do not know of such a book. You can only use your own judgment as to what notes to take during any lecture. "The Nursing Profession : How and Where to Train" might give you some useful information as to filling up your time during the waiting period.

*Agreement.*

(168) I shall be most grateful if you will give me your opinion on this agreement which I was asked to sign after taking up my duties at the end of a week in this parish ?—*A. B.*

You must consult the secretary of the Association.

*Monthly Nursing Certificate.*

(169) We have been trying to place a Jubilee nurse in a very poor district. Since then we have trained women as monthly nurses (not midwives) in the cottages, and on completion of training given them a certificate, which is really a testimonial of good character. Some of the doctors object to this, and say it is illegal for us to grant certificates. Will you kindly tell me if this be so ?—*Questioner.*

You should not call your testimonial a "nurse's certificate," and begin "this is to certify." It is quite misleading. You say that it is really a testimonial of good character ; then why not keep it as such, and nothing more. The form you enclosed is altogether that of a certificate, and there is nothing about "character" in it at all!

**Handbooks for Nurses.**

| | Post Free. |
|---|---|
| "How to Become a Nurse : How and Where to Train." ... | 2s. 4d. |
| "The Nurses' Dictionary" (Pronouncing)... ... ... | 2s. 0d. |
| "Nursing : its Theory and Practice" (Lewis.) ... ... | 3s. 6d. |
| "The Light Treatment" (just published).... ... ... | 2s. 6d. |
| "A Complete Handbook of Midwifery." (Watson). ... | 6s. 4d. |

Of all booksellers or of The Scientific Press, Limited, 28 & 29 Southampton Street, Strand, London, W.C.

### For Reading to the Sick.

"BEARING HIS CROSS."

Up Thy Hill of Sorrows
Thou all alone,
Jesus, man's Redeemer,
Climbing to a Throne:
Thro' the world triumphant,
Thro' the Church in pain,
Which think to look upon Thee
No more again.

Upon my hill of sorrows
I, Lord, with Thee,
Cheered, upheld, yea, carried,
If a need should be:
Cheered, upheld, yea, carried,
Never left alone,
Carried in Thy heart of hearts
To a throne.

*Christina Rossetti.*

Our Lord throughout His earthly life experienced as no other could the isolation of human life; but it is in His Passion that this isolation culminates. He, the Perfect Man who knew no sin, was alone in His Sorrow; "Behold, and see if there be any sorrow like unto My sorrow;" and He was alone in His death. Now, at whatever immeasurable distance this feature of solitude finds its counterpart in every true human life. Think of it, the solitude of human life! We are each one of us alone in our character! Among the myriads of leaves on the trees no two leaves are quite alike; no two among the numberless stones on the seashore are quite alike; no two flowers are quite alike, no two blades of grass. So it is in human life: no two faces are quite alike, so neither are any two characters entirely alike.

Shall we stop for a moment and ask why it is that we find this law of solitude in Nature, whether inanimate or animate, whether irrational or rational? Why is it that my character is different from that of every one else? Why is it that, if my character were perfect, and if others were perfect too, it would still be different from the character of every one else in the world? Why is this?

Why, but because God is infinite and man is finite, and all that one character can do is to exhibit one small ray of the infinite perfection of Almighty God!

My own character, there it stands—different, isolated, alone! Each life that is lived, with its trials and temptations, its opportunities, its successes, its reverses, is in a very real sense alone. God's way in inanimate Nature is to produce harmony out of endless variety; no two blades of grass, no two flowers are alike, but out of this endless diversity there springs a wondrous harmony of form and colour.

So, doubtless, it is intended to be with human life. God's way is to produce harmony out of diversity; myriads of individual characters go to make up the harmony of ordered human life.

If chosen souls could never be alone
In deep mid-silence, open-doored to God,
No Greatness ever had been dreamed or done!

*—Lowell.*

